# Dual-edged swords: a qualitative study of protective and risk factors shaping mental health among Chinese health professions students

**DOI:** 10.1186/s12909-026-10111-2

**Published:** 2026-08-08

**Authors:** Dingke Yu, Wenli Zhu, Linda Dong-Ling Wang

**Affiliations:** 1 School of Basic Medical Sciences & School of Public Health, Faculty of Medicine, Yangzhou University, Yangzhou, 225009 PR China; 2 The First School of Clinical Medicine, Faculty of Medicine, Yangzhou University, 136 Jiangyang Middle Road, Yangzhou, Jiangsu Province 225009 PR China; 3 Present Address: Sau Po Centre on Ageing, The University of Hong Kong, Hong Kong, Hong Kong SAR, China

**Keywords:** Medical education, Mental health, Health professions students, Qualitative study, Bronfenbrenner's ecological systems theory

## Abstract

**Background:**

Mental health challenges among health professions students are a global concern, yet how these challenges manifest within socio-cultural contexts like China remains underexplored. This study utilised Bronfenbrenner’s ecological systems theory to investigate the risk and protective factors influencing the mental health of health professions students in China.

**Methods:**

This qualitative study employed reflexive thematic analysis to examine the experiences and perspectives of 30 undergraduates from clinical medicine, nursing, pharmacy, and allied health programmes. Participants were selected by purposive sampling and data were collected using semi-structured individual in-depth interviews.

**Results:**

During the data analysis, five interconnected themes were identified. Within each theme, participants described factors operating as risks, as protective resources, or as dual-edged forces depending on context. The themes are: (1) Academic Pressure and Interpersonal Dynamics within Immediate Environments (Microsystem); (2) Fragmentation and Dysfunction within Support Networks (Mesosystem); (3) Institutional Logics and Structural Constraints (Exosystem); (4) The Internalisation of Macro-Cultural Values and Behavioural Guidance (Macrosystem); and (5) Chronological Interactions and the Reshaping of Developmental Trajectories (Chronosystem).

**Conclusions:**

The findings reveal a complex transaction across all ecological levels, highlighting that student mental health is predominantly shaped by systemic and cultural forces beyond the individual. This study provides a foundational ecological model for understanding these challenges and suggests that future research quantitatively examine the interactions between these themes, compare experiences across different health disciplines, and evaluate the effectiveness of support interventions tailored to specific ecological levels.

**Supplementary Information:**

The online version contains supplementary material available at https://doi.org/10.1186/s12909-026-10111-2.

## Background

Health professions students worldwide face significant psychological distress due to intense academic workloads, high technical skill demands, and extended training periods [1, 2]. Within this challenging global context, China operates the world’s largest medical education system. While many of these pressures—competitive admissions and long training hours, for example—are shared globally, their local manifestation is shaped by distinctive socio-cultural and institutional forces. These include the Gaokao examination, the widespread expectation of postgraduate training (“forced further study”), and state-mandated rural programmes, all of which may intensify or modify the pressures students experience [3, 4].

In this study, mental health is defined broadly to encompass not only the absence of diagnosable mental disorders (e.g., depression, anxiety), but also the presence of psychological distress (e.g., burnout, stress), alongside help-seeking behaviours and coping resources.

The pervasiveness of these structural pressures is not merely administrative but deeply rooted in Chinese cultural values. Collectivism emphasises family interdependence and social harmony, which translates into strong parental involvement in major life decisions, including career choice [5]. Filial piety—the virtue of respect for one’s parents and ancestors—further reinforces the expectation that children will pursue stable, socially respected professions to bring honour to the family. This cultural backdrop, combined with historical memories of economic instability, fuels a strong societal preference for “iron rice bowl” careers (e.g., medicine, civil service) that offer lifetime security. Consequently, the intense competition and rigid pathways in health education are not arbitrary but reflect a broader cultural logic of risk avoidance and intergenerational obligation.

Beyond academic and structural pressures, it is important to recognise that healthcare work itself involves substantial emotional labour. Caring for patients, particularly those experiencing pain, suffering or death, demands emotional regulation, empathy, and the management of one’s own affective responses [6]. Unlike many other professions with lower emotional demands, repeated exposure to patient suffering has been shown to contribute to compassion fatigue, burnout, and psychological distress among healthcare professionals [7]. Recent evidence indicates that both medical students and nursing students experience compassion fatigue during clinical training, which can affect their well-being and career intentions [8–10]. For these students, these emotional challenges may be particularly salient as they encounter patient suffering before fully developing professional coping strategies, potentially exacerbating their vulnerability to mental health difficulties.

Moreover, students’ perceptions of stress and their coping resources may vary by academic stage: first-year students struggle with the transition to autonomous learning and new social environments, while senior students face the compounded pressures of clinical internships, postgraduate entrance examinations, and imminent career decisions. These developmental differences in maturity and experience may shape how students appraise and manage the demands of their training [11].

Health professions education in China integrates disciplines such as clinical medicine, nursing, pharmacology, and medical technology within comprehensive universities or specialised medical colleges. Undergraduate programmes typically span four to five years, culminating in a mandatory clinical internship year that coincides with intense pressure to pursue postgraduate studies or secure employment [12]. Career pathways are further structured by stringent national licensing examinations and, for medical graduates, a standardised residency training system. Furthermore, state-sponsored initiatives, such as targeted rural medical education programmes, create additional predefined career trajectories for a subset of students. This environment—characterised by shared resources but different career trajectories—creates a distinct ecosystem of pressures for students. Compounding these challenges, China’s substantial population base fosters an exceptionally competitive educational and employment landscape. National data indicate that over half a million medical undergraduates enter the job market annually—a figure that underscores both the scale of the system and the intense competition facing its graduates [13].

Within this high-pressure context, mental health challenges among health professions students have become a critical concern. Evidence indicates that this population experiences markedly higher rates of depression, anxiety, and burnout compared to general undergraduate students [14]. A pre-pandemic meta-analysis reported a depression prevalence of 27.2% among medical students globally, with 11.1% reporting suicidal ideation [15]. The COVID-19 pandemic exacerbated these trends, with multiple studies documenting increased psychological distress, burnout, and suicidal ideation during this period [16, 17]. These difficulties can diminish academic performance, reduce quality of life, and impair professional commitment, potentially contributing to decreased empathy and exacerbated perfectionism or imposter syndrome [18, 19]. As these students represent the future healthcare workforce, their mental health is inextricably linked to future patient care quality and overall health system performance [20].

Understanding mental health in this population requires careful consideration of China’s socio-cultural context. Chinese society is characterised by strong collectivist orientations, where family interdependence and social harmony are highly valued [21, 22]. The COVID-19 pandemic intensified the cultural preference for stability—the avoidance of risk and uncertainty in major life decisions—which manifested as widespread pursuit of state-endorsed, low-risk career paths [23]. While offering security, this preference can create tensions with younger generations’ increasingly diverse aspirations for self-fulfilment.

Many predominant psychological theories, such as Self-Determination Theory, focus primarily on intra-individual processes (e.g., autonomy, competence) and are often applied in Western individualistic contexts [24]. While valuable, these models may not fully capture the systemic and relational forces—such as family expectations, institutional constraints, and cultural norms—that shape Chinese students’ mental health [25]. An individual-level analysis may overlook the ecological layers that constitute significant sources of both stress and support.

To address this gap, our study employs Bronfenbrenner’s ecological systems theory (EST) [26]. Although also developed by a Western psychologist, this framework offers specific strengths for contextualising experiences: it explicitly models multiple interacting levels (micro-, meso-, exo-, macro-, and chronosystem), each of which can capture the kinds of structural and cultural influences that are central to the Chinese context. Unlike theories that focus on individual agency alone, ecological theory allows us to examine how institutional policies (exosystem), cultural values (macrosystem), and temporal changes (chronosystem) intersect with immediate relationships and academic pressures. Thus, EST is not proposed as a “culturally native” theory but as an analytically powerful lens for studying person-context transactions—a need that is particularly acute in collectivist, relationally dense settings such as China.

Guided by this theoretical framework, this qualitative study investigates the protective and risk factors shaping the mental health of Chinese health professions students through in-depth exploration of their lived experiences. The study addresses three research questions: What key factors within the microsystem, mesosystem, exosystem, and macrosystem do students identify as influencing their mental health? How do interactions between these systems shape mental health? And in what ways does the chronosystem—covering critical events such as the COVID-19 pandemic and the changing academic stages—shape these influences over time?

By centring student voices within a systemic framework, this study provides a nuanced understanding of mental health dynamics that moves beyond individual-centred explanations. The findings are expected to contribute to culturally appropriate support systems for health professions students in China, while offering insights for global medical education systems confronting similar ecological challenges.

## Methods

### Study setting and context

A critical contextual factor underpinning this study is China’s national college entrance examination (Gaokao) system. University admission and specific major placement are predominantly determined by Gaokao scores. Students list their preferred majors, but final assignments are highly competitive and based on score rankings and institutional quotas. Those not meeting the cutoff for their first-choice major are often allocated to other programmes within the same institution. This process results in a significant number of students enrolling in majors misaligned with their initial preferences. Although a one-time, highly competitive opportunity to change majors exists after the first year, only a small proportion of students successfully do so.

A particularly salient feature of China’s medical education landscape is the state-sponsored Rural-oriented Tuition-Waived Medical Education (RTME) programme. This initiative is designed to address primary care shortages in rural areas by recruiting students who commit to working in designated township health centres for a specified period after graduation. In return, participants receive a tuition waiver and a modest living stipend during their studies. This state-mandated pathway creates a predefined career trajectory, directly shaping participants’ developmental experiences and distinguishing them from their peers in conventional tracks.

This study was conducted within the medical faculty of a comprehensive public university in Jiangsu Province. The selected university is situated in a region characterised by socioeconomic prosperity alongside challenges such as an aging population and urban-rural healthcare disparities. Its medical curriculum, which integrates rotations in both urban tertiary and rural primary care settings, ensures that students directly encounter these systemic healthcare challenges, providing a rich context for exploring career-related anxieties.

### Sampling strategy and participants

A purposive sampling strategy was employed to capture maximum variation in experiences across key dimensions, including academic major, year of study, and sex (see Table 1). The sampling frame consisted of undergraduate populations of approximately 3,200 in Clinical Medicine, 500 in Nursing, and approximately 200 each in Integrated Traditional Chinese and Western Medicine, Preventive Medicine, Pharmacy, and Medical Laboratory Technology.

Participant recruitment took place between November 2023 and June 2024. Recruitment invitations were distributed during the semesters through class-wide announcements and individual invitations.

Health professions education in China follows structured programmes, typically spanning four years (e.g., Pharmacy, Nursing) or five years (e.g., Clinical Medicine, Preventive Medicine). The sample intentionally included students from both programme lengths. To capture the variation within the broad field of clinical medicine, the sample included students oriented from the undergraduate level towards specific specialties such as Obstetrics and Gynaecology and Paediatrics, who complete the same core curriculum as general clinical medicine students. To include illustrative examples of predefined career tracks, the sample also included two students enrolled in the RTME programme.

### Data collection

Data were collected through semi-structured, individual in-depth interviews. These interviews explored the complex factors influencing the mental health of Chinese health professions students. The semi-structured interview guide (see Supplementary Material) was developed specifically for this study, based on a systematic review of existing literature and structured using EST as a framework. It contained open-ended questions designed to elicit participants’ experiences across all five levels of the model: the microsystem (e.g., academic pressure, interpersonal relationships), mesosystem (e.g., home-school connections), exosystem (e.g., job market, institutional systems), macrosystem (e.g., cultural values), and chronosystem (e.g., key life events). Flexible probing techniques were used to ensure comprehensive coverage of the theoretical framework while allowing participants’ unique perspectives to emerge.

To ensure ethical integrity and data reliability, several measures were implemented. Prior to each interview, the researcher detailed the study’s purpose, confidentiality procedures, and the participants’ right to withdraw, after which written informed consent was obtained. All interviews were conducted by a researcher (DKY, female) who had no teaching or grading relationship with the participants and had received professional training in qualitative methods. This approach minimised power dynamics and fostered a safe environment for open communication. Interviews were held in a private university room, with one exception, conducted via secure video call for a student on internship. Each session lasted approximately 60 min and was audio-recorded in full, with the interviewer also taking notes. Upon completion, participants were compensated with a small gift (valued at approximately 10 CNY) for their time.

### Data processing and analysis

This study employed a constructivist paradigm and Braun and Clarke’s reflexive thematic analysis [27]. EST served as a sensitising framework [28] to inform the interview guide and organise the interpretation of findings, rather than a predetermined coding framework applied to the data.

Audio recordings were transcribed verbatim. A trained transcriber (WLZ) produced the initial transcript, which was then verified sentence-by-sentence against the audio by another researcher (DKY). All transcripts were de-identified, assigned unique codes (S1-S30), and managed using NVivo 20 software (QSR International). Two core researchers (DKY, WLZ) conducted the analysis collaboratively, with regular input from the senior researcher (LDLW). Coding was inductive and data-driven [29, 30]: open codes emerged directly from participants’ narratives without imposing theoretical categories. The researchers coded independently, compared their codes, negotiated consensus, and developed a codebook. Related codes were then grouped into candidate themes. These themes were iteratively reviewed and refined in dialogue with EST.

Data collection and analysis proceeded iteratively. After every 5–6 interviews, the team (DKY, WLZ, LDLW) met to discuss emerging concepts, refine subsequent interviews, and assess data richness. We adopted code saturation [31] as a practical criterion to determine sample size: data collection continued until no new codes emerged. After 27 interviews, preliminary analysis indicated thematic stability; three additional interviews (S28-S30) confirmed saturation, yielding no new codes. Recruitment ceased at 30 participants.

### Reflexivity statement

The research team comprised one faculty member (LDLW) and two postgraduate researchers (WLZ, DKY). To mitigate potential power dynamics, the faculty member was not involved in participant recruitment or data collection. All interviews were conducted solely by DKY who had no prior teaching or assessment relationship with participants.

We engaged in ongoing reflexivity through team discussions after every 5–6 interviews, examining how our professional backgrounds (clinical medicine, public health) and personal experiences as former health professions students might shape our interpretations. We documented these reflections in a team reflexivity journal and revisited them during coding to ensure interpretations remained grounded in the data.

The two postgraduate researchers maintained individual notes on emerging interpretations. Coding disagreements were resolved through collective discussion, with the faculty member consulted when needed. An audit trail documented all decisions.

All data analysis was conducted using the original Chinese transcripts. Quotations were translated into English by a bilingual researcher (DKY) and independently verified against the original Chinese by a second researcher (WLZ); discrepancies were resolved by team consensus.

### Ethical approval

This study received ethical approval from the Research Ethics Committee of the University with which the authors are affiliated (YXYLL-2023-155), ensuring informed consent of participants, privacy protection, and data anonymisation. This study adhered to the Consolidated Criteria for Reporting Qualitative Research (COREQ) checklist [32].

## Results

**Fig. 1:**
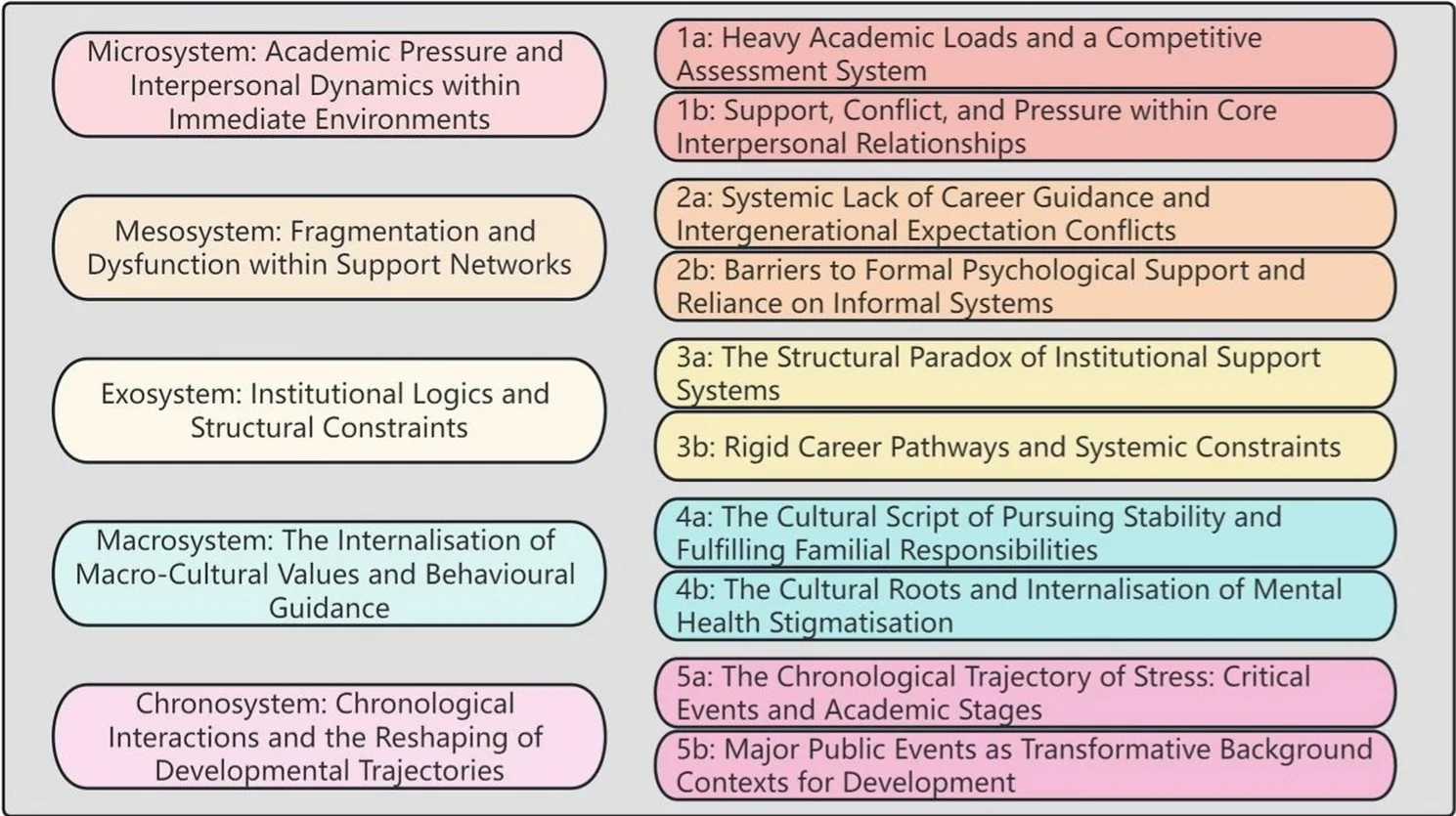
Themes and subthemes

We conducted semi-structured, individual in-depth interviews with 30 health professions students (see Table 1 for demographics). A reflexive thematic analysis, using EST as a sensitising framework to organise and interpret findings, identified five overarching themes, each with two subthemes (see Fig. 1): (i) Academic pressure and interpersonal dynamics within immediate environments (Microsystem); (ii) Fragmentation and dysfunction (Mesosystem); (iii) Institutional logics and structural constraints (Exosystem); (iv) The internalisation of macro-cultural values and behavioural guidance (Macrosystem); and (v) Chronological interactions and the reshaping of developmental trajectories (Chronosystem). In the findings below, we describe how participants experienced various factors as operating as risks, as protective resources, or as dual-edged forces depending on context.

**Table 1:** Basic information of participating students (*N* = 30)

Category		Number of students (*n*)	Percentage (%)
Sex			
	Male	15	50.0%
	Female	15	50.0%
Major			
	CM^1^	11	36.6%
	Pharmacy	6	20.0%
	MLT^2^	4	13.3%
	CM (targeted)	2	6.6%
	Nursing	2	6.6%
	PM^3^	2	6.6%
	TCM&CL^4^	1	3.3%
	CM (Obstetrics and Gynaecology)	1	3.3%
	CM (Paediatrics)	1	3.3%
Grade			
	Year-1	6	20.0%
	Year-2	12	40.0%
	Year-3	5	16.6%
	Year-4	4	13.3%
	Year-5	3	10.0%
Age			
	18–20	21	70.0%
	21–24	9	30.0%
Total		30	100.0%

*CM* Clinical medicine, *MLT* Medical laboratory technology, *PM* Preventive Medicine, *TCM&CL* Integrated Traditional Chinese and Western Medicine

### Theme 1: microsystem: academic pressure and interpersonal dynamics within immediate environments

This theme captures the factors within students’ most immediate environments that shape their psychological health, primarily characterised by intense academic demands and complex interpersonal relationships.

#### Subtheme 1a: heavy academic loads and a competitive assessment system

Participants described a demanding academic environment defined by voluminous coursework, rigorous content, and frequent high-stakes examinations. A pervasive competitive culture—evident in grade rankings, scholarship competition, and the pursuit of postgraduate recommendations—functioned as a direct and chronic source of psychological stress. This pressure was not merely about passing courses but about outperforming peers in a system where future careers depend on relative standing.

The intensity of this pressure became most visible during examination periods, when students reported normalising extreme behaviours. One fifth-year clinical medicine student (obstetrics and gynaecology track) explained:

“Especially when it comes to final exams … it’s exhausting. Everyone is staying up all night … it’s common to pull all-nighters right before exam week.” (S26, CM (Gynaecology), Year-5).

This statement illustrates how observing peers’ study habits in the immediate environment—“everyone is staying up”—can normalise sleep deprivation, turning occasional late nights into an expected practice. The cumulative effect of such sustained pressure over years of training led students to question whether the personal cost was worth the professional reward. Another fifth-year clinical medicine student reflected:

“After five years of study, I think studying medicine is indeed very hard … If someone asked me if I would recommend studying medicine? I’d honestly say I do not recommend it.” (S23, CM, Year-5).

This reflection captures a tension between continued commitment to medicine and an honest recognition of its toll—exhaustion, sacrificed personal time, and the weight of years of pressure. The student does not reject medicine as a career but acknowledges the extraordinary personal cost.

For participants who engaged in scientific research, academic pressure was compounded by self-initiated activities. A second-year preventive medicine student explained:

“I feel the pressure in the research group is greater than academic pressure … For the past three or four months, I basically attend classes during the day, return in the evening and start reading … busy until 12 midnight every day.” (S30, PM, Year-2).

This quote shows that pressure from voluntary research involvement can exceed that from required coursework, suggesting that students internalise expectations for research productivity even when not formally mandated.

The peer learning environment acted as a dual-edged sword. On one hand, it provided crucial solidarity. The same fifth-year clinical medicine student (obstetrics and gynaecology track) noted:

“The atmosphere in our class and our dormitory is quite good; everyone is studying together … Sometimes we roommates also encourage each other and share our feelings.” (S26, CM (Obstetrics and Gynaecology), Year-5).

Here, peer support functions as a protective factor—a collective coping resource that buffers against individual distress. On the other hand, the same peer environment generated implicit pressure through constant social comparison. A third-year clinical medicine student recounted:

“When I first entered my freshman year … my roommate studied until 12 every night. Then I couldn’t sleep in bed, so I got down to study too.” (S1, CM, Year-3).

This example illustrates how social comparison in the immediate environment—observing a roommate’s visible effort—can shape a student’s own behaviour, not because of internal motivation or course demands, but because of the perceived need to match peers. Within the microsystem, academic pressure thus operates as a sustained, multi-layered risk factor that erodes well-being while occasionally offering protective peer support.

#### Subtheme 1b: support, conflict, and pressure within core interpersonal relationships

In interviews, participants described interpersonal relationships as a dual-edged domain. The same relationships—with roommates, peers, teachers, and family—could be sources of both psychological support and distress, depending on the context and the quality of interaction.

Peers were described as an emotional resource. A first-year medical laboratory technology student explained her coping strategy:

“I write in my diary first, then go chat with good friends. And also my parents, my mom.” (S18, MLT, Year-1).

This sequence—diary first, then friends, then family—suggests that students turn to self-regulation before seeking social support, with peers playing a subsequent but still important role.

However, participants also noted that university friendships felt different from those formed in high school. One second-year clinical medicine student observed that friendships at university seemed more superficial than the deep, ‘heart-to-heart’ connections of high school, describing them instead as instrumental ‘meal buddies’ *(S7*,* CM*,* Year-2).* This comparison reveals an unmet expectation: students anticipate deep emotional bonds but encounter relationships organised around convenience and shared meals. The term “meal buddies” captures a certain practical, non-intimate quality that contrasts with the support students had previously relied on.

Dormitory relationships added another layer of complexity. Participants described them as generally manageable, but differences in daily habits—sleep schedules, noise levels, cleanliness—could generate persistent tension. In one account, a second-year nursing student reported that while conflicts were usually resolved through communication, ongoing issues led to a tense living environment, and she described the dormitory atmosphere as ‘very oppressive’ *(S29*,* Nursing*,* Year-2)*. The word “oppressive” is notable: it suggests that unresolved dormitory conflicts can permeate the student’s daily environment, affecting rest, study, and emotional well-being. Here, the same relationships that could offer support instead become a chronic stressor.

Interactions with faculty were predominantly task-oriented. A first-year clinical medicine student admitted:

“Actually, I don’t really like asking teachers questions … I’m afraid the teacher might think I’m foolish.” (S4, CM, Year-1).

This statement reveals unease about approaching teachers, rooted not only in fear of being judged as incapable but also in the perceived authority of the teacher. The student refrains from seeking academic help despite likely needing it, transforming a potential support interaction into a risk-avoidance behaviour. Such hesitation may limit students’ access to faculty mentorship, an otherwise valuable resource.

Family relationships were described as a foundational source of support, yet they could also carry past strain. One third-year clinical medicine student shared:

“My dad has a bit of a temper; we generally don’t talk to him much … he was abusive, so we are quite afraid of him.” (S1, CM, Year-3).

This account shows that family support is not automatically available; past experiences of harsh treatment and ongoing fear can sever the very channel that students most need in times of distress. The student does not describe seeking help from this parent, highlighting how prior family interactions shape current help-seeking behaviour.

Across these accounts, interpersonal relationships within the microsystem functioned as a domain where protective and risk dynamics coexisted. Peer and family connections offered vital emotional resources, but when expectations of intimacy were unmet, conflicts arose, or power imbalances inhibited help-seeking, the same relationships became sources of distress. Thus, the microsystem’s relational environment is not uniformly supportive or harmful; its impact depends on the specific quality of each interaction and the student’s perception of safety and reciprocity.

### Theme 2: mesosystem: fragmentation and dysfunction within support networks

This theme identifies the disconnections and functional failures within the support networks linking family, school, and peers. These mesosystemic weaknesses manifested as a systemic lack of career guidance and barriers to accessing formal psychological support.

#### Subtheme 2a: systemic lack of career guidance and intergenerational expectation conflicts

Participants pointed to a critical gap in systematic career guidance at two key junctures: selecting a university major during the Gaokao process and planning for future careers. This absence left students making high-stakes decisions with limited information, resulting in passive major assignments and a tension between familial expectations of “stable” careers and students’ own interests.

A first-year medical laboratory technology student described the confusion surrounding her major choice:

“The first three preferences were filled in randomly … I was in a complete mess … They (parents) didn’t know either, so they just asked relatives.” (S18, MLT, Year-1).

This account illustrates how a lack of structured guidance can push students and families into reliance on informal advice from relatives—advice that may be well-intentioned but not necessarily informed about current labour market conditions or the student’s aptitude. The student’s phrase “a complete mess” reflects the emotional turmoil of being unprepared for a decision that would shape her entire educational trajectory. Another third-year pharmacy student discovered only after enrolment that his major was considered “unpopular” *(S13*,* Pharmacy*,* Year-3)*, a label that later intensified his anxiety about job prospects. The term “unpopular” here signals not just a matter of prestige but a perceived mismatch between supply and demand in the job market. This account illustrates how students can commit to a career pathway with limited understanding of its long‑term labour market outcomes, especially when career guidance from both family and school is absent. This gap underscores the difficulty of making informed decisions without coordinated support across different life contexts.

Family pressure to pursue “stable” professions—medicine, teaching, civil service—was evident. A first-year clinical medicine student recounted how his parents overrode his preferences:

“They (parents) insisted I list that thing (university major), I just didn’t want to study it … There were some subtle arguments … My mom … couldn’t offer any … better advice.” (S4, CM, Year-1).

The hesitation and ellipses in this quote (“couldn’t offer any … better advice”) suggest that even when parents exert strong influence, they may themselves be uncertain about what constitutes “better”. Parental insistence is not necessarily grounded in superior knowledge; it often reflects a culturally ingrained belief that certain careers guarantee security. This dynamic creates a double bind for students: they are expected to follow parental directives while simultaneously bearing the consequences if those directives lead to dissatisfaction.

In certain accounts, this intergenerational conflict led students to abandon their own aspirations altogether. A second-year clinical medicine student in the RTME programme stated:

“It wasn’t my choice back then; I was tricked into coming here by my dad … He always wanted me to study medicine … I personally wanted to study Physics and Philosophy.” (S11, CM (targeted), Year-2).

The word “tricked” carries strong emotional weight, implying deception and betrayal rather than mere disagreement. This participant’s original interest in Physics and Philosophy represents a very different academic and career path. His sense of being trapped in a major not of his choosing continued well into his second year, suggesting that unresolved career misalignment does not simply fade with time.

For those assigned to non-preferred majors, the possibility of changing majors existed but was perceived as blocked by “sunk cost”—time already invested—and the procedural complexity of transferring. Participants described a sense of resigned persistence, continuing in a field they did not choose rather than facing the perceived costs of switching.

From a risk and protective perspective, the systemic lack of career guidance operated as a mesosystemic risk factor. The absence of effective linkage between family expectations, school counselling, and students’ own exploration created a vacuum filled by uninformed advice and cultural pressure.

#### Subtheme 2b: barriers to formal psychological support and reliance on informal systems

When facing psychological distress, students described relying on informal networks such as friends and family. The university’s formal psychological support system was described with perceived limitations that could discourage help-seeking.

One perceived limitation was a fundamental lack of awareness that these services existed. A preventive medicine student expressed:

“I haven’t even heard of a psychological service station … I don’t even know who the psychological committee member is … He seems to have never posted any information.” (S27, PM, Year-2).

This account points to a gap in basic service outreach, where students cannot access resources they are unaware of. The student’s reference to the “psychological committee member” suggests that even when peer support roles are formally assigned, they may be functionally invisible to the general student body.

Even when students were aware of the service, they expressed deep skepticism about its effectiveness, viewing it as unable to address root causes and sometimes dismissing it as generic “nonsense”. One participant explained that formal help was seen as unable to address the underlying sources of their distress.

Furthermore, the methods of service promotion and access were described as insufficient and intimidating. Participants suggested that the requirement for in-person visits deterred those in need, recommending more discrete and accessible options such as peer-led support hotlines. Participants appeared unaware of existing alternative formats (e.g., online chat services), suggesting that even when such options exist, lack of awareness renders them unavailable.

Students used self-regulation or informal social networks for support. In interviews, participants described various coping strategies, including sharing emotions with close friends and self-regulation.

In this subtheme, perceived limitations of the formal psychological support system functioned as mesosystemic risk factors. Participants relied on informal networks and self-regulation for support.

### Theme 3: exosystem: institutional logics and structural constraints

This theme focuses on the broader institutional and environmental structures that students do not directly control but which profoundly shape their experiences. The analysis reveals a structural paradox within institutional support systems and the rigid, external constraints that define career development pathways.

#### Subtheme 3a: the structural paradox of institutional support systems

Participants reported that the university’s institutional systems, ostensibly designed to support them, paradoxically created difficulties for students in practice. Due to inherent design flaws, these systems intended to provide resources inadvertently created difficulties for students.

This paradox was evident in outdated infrastructure that compromised both learning experiences and safety. Students cited aging laboratory equipment and malfunctioning classroom technology as sources of frustration and risk. The recurrence of these technical failures—despite their seemingly minor nature—signals a broader institutional neglect that undermines students’ trust in the learning environment.

Inefficient course scheduling and management processes further highlighted this misalignment. Students described chaotic curriculum integration and impractical logistics, such as being scheduled for back-to-back classes on different campuses, which added strain to their daily lives. One third-year clinical medicine student explained:

“It feels like the course scheduling means one course has many different teachers teaching it, and each teacher covers different content, making it hard to grasp the key points … When it comes to revision, we have to reorganise everything ourselves; it’s particularly chaotic.” (S1, CM, Year-3).

This account illustrates how administrative decisions, such as dividing a single course among multiple instructors without coordination, transfer the burden of integration onto students. Instead of receiving a coherent curriculum, students must piece together disparate content during revision, a task that adds cognitive load and frustration to an already demanding schedule.

Furthermore, certain administrative measures were perceived as rigid and formalistic, ultimately adding to student burden rather than alleviating it. A first-year pharmacy student who served as a student cadre criticised mandatory morning routines:

“We have morning self-study plus morning runs … After the morning run, plus a morning reading session and the first class, I’ve observed the students’ state—it’s detached … This kind of formalistic arrangement actually makes everyone more tired.” (S14, Pharmacy, Year-1).

The student’s observation that classmates appear “detached” after these required activities reveals a perverse outcome: measures intended to promote discipline and health instead exhaust students, reducing their ability to engage in the first class of the day. The term “formalistic arrangement” suggests that such policies are followed mechanically without regard for their actual effects on student well-being.

Even detail-level issues in student services, such as the lack of control over classroom air conditioning temperature, reflected a systemic disconnect between institutional operations and student needs. Competitive incentive mechanisms, such as guaranteed postgraduate recommendations, were described as demotivating for students who perceived the benchmarks as unattainably high, creating a sense of resignation rather than encouragement. One participant noted that knowing the criteria were out of reach led to disengagement rather than extra effort—an unintended consequence of a system designed to reward excellence.

From a risk and protective perspective, the structural paradox of institutional support systems, including outdated infrastructure, inefficient scheduling, and rigid administrative measures, functioned as exosystemic risk factors.

#### Subtheme 3b: rigid career pathways and systemic constraints

Career development in the health professions was described by participants as a rigid, linear path, strictly governed by external labour market demands. This manifested as a mandatory requirement for advanced degrees—a phenomenon participants described as “degree inflation”—which triggered intense competition and shaped student trajectories from the outset.

A fifth-year clinical medicine student articulated this reality:

“The current major context of medicine is that if you don’t have a master’s degree, or even a doctoral degree, it’s very difficult to find a job in a large tertiary Grade A hospital … This is forced by the employment situation.” (S23, CM, Year-5).

The student’s phrasing “forced by the employment situation” suggests that postgraduate study is experienced as an externally imposed necessity, not merely a voluntary career aspiration. The job market, rather than intrinsic interest, shapes the perception of what step is required next.

This systemic requirement made postgraduate study seem like an inevitable and often personally misaligned path. Participants described the pressure as intense, with students feeling they had “no other way” and that failing to pursue further education equated to unemployability. A third-year pharmacy student explained the stark salary differential:

“The salary for postgraduate and undergraduate students is completely on different levels … Undergraduate students can only do some manual labour … There’s no upward mobility.” (S13, Pharmacy, Year-3).

The phrase “no upward mobility” captures a sense of career stagnation without an advanced degree. This perception is not limited to clinical medicine; it extends to other health professions, creating anxiety about market saturation and limited prospects. The normalisation of the master’s degree as a basic entry requirement—what participants termed “forced further education”—has permeated even primary-level medical units.

For participants in the RTME programme, career pathways were pre-determined by state mandate. One RTME student noted that this path exempted them from the pressure to pursue postgraduate studies:

“We don’t need to … consider postgraduate entrance exams; our study pressure is much lighter.” (S10, CM (targeted), Year-2).

This account illustrates how state-mandated career tracks can reduce competition pressure for some students. However, for another RTME student whose personal ambitions were misaligned with the programme, the contractual obligation became a source of constraint:

“It’s boring to stay in the community … As for my career, I definitely want to go to a big city … But if you breach the contract … first of all, the money must be paid back … and it will be recorded as a default … because the contract has already been signed.” (S11, CM (targeted), Year-2).

The student’s repetition of consequences—“money must be paid back”, “recorded as a default”, “contract has already been signed”—conveys a sense of being trapped. The security of guaranteed employment comes at the cost of career autonomy, creating a central tension between stability and aspiration.

From a risk and protective perspective, rigid career pathways operated as an exosystemic risk factor, intensifying competition and narrowing perceived options. The RTME programme thus operated as a dual-edged force: for the participant who valued reduced postgraduate pressure, it functioned as a protective factor; for the participant who experienced contractual constraint on career autonomy, it functioned as a risk factor.

### Theme 4: macrosystem: the internalisation of macro-cultural values and behavioural guidance

This theme reveals how macro-level socio-cultural values are internalised by students, profoundly shaping their career choices, perceptions of psychological health, and help-seeking behaviours.

#### Subtheme 4a: the cultural script of pursuing stability and fulfilling familial responsibilities

Within the Chinese socio-cultural context, “stability” emerged as a central tenet of career choice. This value is deeply rooted in historical memory and regarded as essential for mitigating life risks and ensuring long-term family security. Selecting a “stable” career path was described not merely as an individual decision but as a culturally sanctioned act of fulfilling family responsibility and bringing honour to the family. This cultural script, intertwining the pursuit of stability with the fulfillment of family obligations, shaped participants’ major selection, with medicine seen as an ideal vehicle to realise these dual objectives.

A second-year preventive medicine student explained how parental expectations guided her choice:

“It was mainly that my parents thought maybe, either apply for normal school (to become a teacher) or apply for clinical medicine … Their feelings were the main thing … If it’s something parents really want, then you ultimately won’t go against their wishes.” (S30, PM, Year-2).

This account illustrates how filial deference operates: the student subordinates her own preferences to her parents’ desires, framing compliance as the natural, expected response (“you ultimately won’t go against their wishes”). The phrase “their feelings were the main thing” suggests that emotional obligation outweighs personal interest in the decision-making hierarchy.

Personal interest could also align with the cultural ideal of an “iron rice bowl”—a secure, lifetime job with guaranteed stability and benefits. A first-year pharmacy student described how his interest in biology merged with the pursuit of socio-economic security:

“The idea of becoming a doctor germinated from my fondness for biology … and the first thing I thought of was an ‘iron rice bowl’… It involves biology … and it allows you to save people …” (S14, Pharmacy, Year-1).

The student’s phrasing “the first thing I thought of” reveals that stability was not an afterthought but a primary consideration, seamlessly integrated with intrinsic interest. For him, the cultural script of stability and personal passion were not in conflict but mutually reinforcing.

This “stability-first” value also extended to postgraduate planning. Participants described pursuing advanced degrees as the next logical step in a socially recognised path to a secure career, further influenced by continued family expectations.

From a risk and protective perspective, the cultural script prioritising stability and family responsibility operated as a dual-edged force. For participants whose interests aligned with this script, it provided a clear, socially sanctioned path and a sense of security (protective). For participants whose personal aspirations diverged from this script, it created internal conflict and constrained autonomy (risk).

#### Subtheme 4b: the cultural roots and internalisation of mental health stigmatisation

Students’ attitudes towards mental health were deeply influenced by traditional cultural values emphasising “toughness,” “self-control,” and the preservation of “face” (personal dignity and social reputation). In participants’ accounts, these values were associated with a preference for self-reliance and a reluctance to seek formal help.

A third-year pharmacy student described the expectation of self-reliance:

“Generally, when encountering any difficulty, I solve it myself … self-regulation … making peace with myself, slowly adjusting … you just need to think it through yourself.” (S13, Pharmacy, Year-3).

This account illustrates the internalisation of “toughness”: the student frames mental health as a matter of individual willpower (“think it through yourself”), with no mention of external support. The repetition of self-directed actions (“solve it myself”, “self-regulation”, “making peace with myself”) reinforces a narrative of self-reliance.

This preference for self-reliance rendered formal psychological support as a secondary or last resort. Participants expressed a belief that their problems were not “serious enough” to warrant professional intervention. A first-year pharmacy student explained:

“When I encounter these problems, I definitely prioritise self-regulation. Our university has psychological counselling service, right? I haven’t been there yet. If I really encounter serious issues, I could go and ask.” (S12, Pharmacy, Year-1).

The conditional phrasing “If I really encounter serious issues” reveals an implicit threshold: only extreme distress justifies professional help. Everyday struggles, even if persistent, are relegated to self-management.

A second-year nursing student explicitly linked embarrassment to the decision not to seek help:

“I’d feel somewhat embarrassed … I feel it probably isn’t serious enough to that extent.” (S29, Nursing, Year-2).

Here, embarrassment acts as a direct deterrent, compounded by the perception of insufficient seriousness. The hesitation in the quote (“somewhat embarrassed”, “probably isn’t”) suggests uncertainty, yet the combined effect creates a powerful barrier.

The concept of “face” emerged as an underlying force, fostering fear that disclosing vulnerability would lead to negative social evaluation. A fourth-year clinical medicine student observed:

“I feel there are quite a few people in medical school with poor psychological states, but I don’t get the sense they have the idea of seeking outside help … they always feel it’s nothing major, shouldn’t trouble the teacher … feeling that seeking out a teacher must be for bad things.” (S24, CM, Year-4).

This observation captures a collective perception: help-seeking is associated with “bad things”—implying serious pathology or shame. The student’s phrasing “shouldn’t trouble the teacher” reveals a norm of not burdening authority figures with personal problems, reinforcing the barrier to accessing formal support.

From a risk and protective perspective, the internalisation of mental health stigmatisation functioned as a macrosystemic risk factor that suppressed help-seeking. Self-reliance and self-regulation were described by participants as coping strategies, but these also reflected internalised cultural norms that could prevent access to professional support when needed.

### Theme 5: chronosystem: chronological interactions and the reshaping of developmental trajectories

This theme elucidates the temporal dimension of students’ mental health, revealing it as a dynamic state that evolves in response to key life events, distinct academic stages, and major public events.

#### Subtheme 5a: the chronological trajectory of stress: critical events and academic stages

Participants’ accounts of psychological stress revealed a dynamic pattern over time, shaped by the interplay of acute critical events and the evolving demands of different academic stages. Discrete negative events—such as academic failure or interpersonal conflict—could trigger significant and long-lasting distress, while the transition from early to later training years introduced qualitatively different stressors.

Past academic setbacks continued to resonate years after they occurred. A second-year nursing student recounted:

“I could have gotten into the best high school in our area, the key high school, but I fell short by one point, and then went to the second high school. I feel that was the biggest setback in my life … I was sad throughout the entire first year of high school and didn’t recover from it.” (S28, Nursing, Year-2).

This account illustrates how a single academic setback can dominate a student’s emotional experience for an extended period, in this case throughout the first year of high school.

Similarly, poor performance on practice postgraduate entrance exams could provoke acute crises of confidence. A fourth-year pharmacy student described:

“Everyone was preparing for the postgraduate exam, but then I got only 50 points in English I [on a practice test]. I was crushed then … I cried at that time, called my mom and said I’m not taking the postgraduate exam anymore. Felt like I had studied for so long, yet still only scored 50 in English, was really upset then.” (S21, Pharmacy, Year-4).

The intensity of the reaction—“crushed”, crying, calling a parent to announce abandonment of the exam—reveals how a single poor performance can threaten a student’s sense of academic competence and future plans. The student’s emphasis on having “studied for so long” highlights the perceived waste of effort, compounding the distress.

Beyond academics, painful interpersonal events, including family conflicts and past experiences of bullying, were reported as formative traumas that contributed to psychological vulnerability during university. A second-year medical laboratory technology student shared:

“In junior high, it was kinda school bullying, cyberbullying. After the senior high school entrance exam ended … it became like the whole class was there cursing … it did affect me a lot … that emotional breakdown I had in the first semester of freshman year was actually related to that.” (S17, MLT, Year-2).

This account shows how bullying experiences in junior high continued to affect the student years later, contributing to an emotional breakdown during the first semester of university. The persistence of such distress highlights the long-term psychological impact of early interpersonal trauma.

Superimposed on these acute stressors was a background of evolving academic demands. Early-year students described challenges in adapting to more autonomous learning modes, struggling to transition away from the “cramming-style” pedagogy of secondary school. Senior students, in contrast, described the compounded pressures of full-time clinical internships and preparation for postgraduate entrance examinations, with participants describing this period as the most difficult of their studies.

From a risk and protective perspective, critical negative life events (academic setbacks, exam failures, interpersonal trauma) functioned as chronosystemic risk factors that could have lasting effects on mental health. The evolving demands of different academic stages added chronic stressors, with the combination of clinical internships and postgraduate exam preparation in the senior year described as particularly intense.

#### Subtheme 5b: major public events as transformative background contexts for development

The COVID-19 pandemic functioned as a major systemic event whose impact extended beyond a discrete stressor. It altered learning environments, social patterns, and career perceptions, becoming a persistent background condition that reshaped developmental trajectories.

The pandemic directly influenced students’ perceptions of their fields of study and the job market. A second-year preventive medicine student noted:

“I feel employment might be quite difficult … during the pandemic, the CDC [Chinese Center for Disease Control and Prevention] recruited many, many people … feels like compared to other majors, the market might be more saturated.” (S30, PM, Year-2).

This account illustrates how pandemic-related expansion of certain public health roles led the student to anticipate future job market saturation—a perception that added career anxiety despite the field’s apparent growth. The repetition of “many, many people” emphasises a perceived oversupply.

The health crisis also reinforced the macrosystemic cultural preference for “stable” careers. Economic uncertainty appeared to strengthen familial pressure to pursue state-sanctioned professions, which were seen as secure “iron rice bowls”. One participant noted that the post-pandemic environment made careers such as teaching or medicine appear as “the most reliable” paths to stability.

Pedagogically, the shift to online learning presented adaptation challenges. Students reported decreased engagement and motivation during remote classes, contrasting the remote format with the in-person classroom, where the presence of peers and instructors was described as fostering greater focus and discipline.

Furthermore, the pandemic’s disruption of the labour market intensified the perceived necessity of postgraduate degrees. Students observed that the job market for undergraduates had become “saturated”, solidifying the postgraduate entrance examination as an “unavoidable” step in their career pathway.

From a risk and protective perspective, the COVID-19 pandemic operated as a transformative chronosystemic event that amplified existing macrosystemic pressures (career stability preference) and introduced new stressors (online learning challenges, labour market disruption). Students in fields like preventive medicine perceived pandemic-related expansion of their discipline, though this also led to concerns about market saturation.

## Discussion

This qualitative study, guided by EST, provides a nuanced understanding of the multifaceted factors shaping the mental health of health professions students in China. Our analysis revealed five interconnected themes, illustrating the complex interplay between individual experiences and broader systemic influences across the ecological landscape.

Synthesis of risk and protective patterns. When synthesising the findings, the following patterns emerged from participants’ accounts. Risk dynamics appeared in accounts of: heavy academic loads and competitive assessment (microsystem); fragmentation of support networks and barriers to formal psychological help (mesosystem); rigid career pathways (“forced further study”) and paradoxical institutional policies (exosystem); internalised mental health stigmatisation (macrosystem); and cumulative critical life events (chronosystem). Protective dynamics appeared in accounts of: supportive peer and family relationships (microsystem); informal coping networks (mesosystem); for RTME students, exemption from postgraduate entrance pressure (exosystem); and collective study atmospheres (microsystem). Participants described factors as dual-edged in their accounts: family expectations provided security but constrained autonomy; peer environments fostered solidarity but intensified social comparison; institutional incentives were described as motivating in some accounts and demotivating in others. This complexity supports our contention that an ecological systems approach offers a more nuanced understanding than a simple risk/protective dichotomy.

### Microsystem: academic pressure and interpersonal dynamics within immediate environments

Our findings illuminate the immediate environment where students’ mental health is most directly challenged. Academic life and core interpersonal relationships function as a dual-edged sword, simultaneously constituting primary sources of stress and essential buffers, aligning with Bronfenbrenner’s emphasis on the profound impact of proximal processes [26].

Participants described an academic environment characterised by voluminous coursework and frequent high-stakes examinations. A highly competitive assessment system, manifested through grade rankings and the intense competition for postgraduate recommendations, created a pervasive climate of chronic stress. This finding is consistent with previous research, which has identified academic pressure as a common stressor among health professions students [33]. However, our study contextualises this pressure within the Chinese system, where pursuing a master’s or doctoral degree is a non-optional prerequisite for a viable career path—a phenomenon termed “forced further study”. This transforms a developmental milestone into a significant and persistent stressor, potentially fuelling burnout and disengagement.

Furthermore, the peer environment functioned as a dual-force. A collective study atmosphere and supportive peer relationships were crucial protective factors. Conversely, the same environment exacerbated pressure through constant social comparison, with students feeling compelled to match peers’ intensive study habits. This aligns with social comparison theory, where evaluating one’s efforts against a reference group of relentlessly exerting classmates can be detrimental to well-being [34, 35].

The critical, yet complex, role of interpersonal relationships is evident. While strong connections with roommates, peers, and family served as vital buffers against academic stress [36], our data highlight complexities within the Chinese boarding university context. Frictions from shared living spaces represented a persistent low-grade stressor. More significantly, we identified a reported shift from deep, “heart-to-heart” friendships in high school to more instrumental “meal buddy” relationships in university. This attenuation of a key support mechanism during a period of heightened stress may intensify feelings of isolation, a known risk factor for mental health difficulties [37].

Students-faculty dynamics also emerged as distinctive. The reported hesitancy to approach teachers due to fear of being perceived as “foolish” reflects a perceived power distance and cultural emphasis on teacher authority [38]. This barrier limits access to informal academic and emotional support from instructors, a resource considered vital in educational studies emphasising faculty mentorship [39, 40]. In contrast, family relationships were predominantly described as supportive, yet they could also be a source of conflict or historical trauma [41], underscoring that the family microsystem can carry pre-existing strains into the university experience [42].

### Mesosystem: fragmentation and dysfunction within support networks

Our analysis reveals critical disconnections between the microsystems of family, school, and peers. Instead of forming an integrated support network, these fragmented connections often exacerbate distress, highlighting a failure in the ecological transitions between students’ key life contexts [42, 43].

One mesosystemic failure is the absence of effective guidance at the critical juncture between secondary and higher education. Participants made pivotal decisions about their majors with minimal professional support, relying on familial advice heavily weighted towards societal perceptions of stability [5, 44, 45]. This leads to a passive major selection, resulting in what we term professional identity dissonance—a persistent conflict between personal interests and imposed career paths [46]. Students in non-preferred majors expressed a lack of belonging and diminished motivation, resonating with literature on poor person-environment fit [47]. The cultural imperative to fulfill family expectations, combined with perceived “sunk costs” [48] and administrative hurdles, creates powerful mesosystemic barriers, demonstrating a failure to mediate constructively between family and academic systems [49].

The university’s formal psychological support system also presented structural and operational barriers that disrupted its connection to students [50]. Participants reported a triad of access issues: (1) a fundamental lack of awareness due to insufficient outreach [51]; (2) procedural inaccessibility, such as in-person visits heightening privacy concerns [52]; and (3) a perceived lack of efficacy, with services viewed as offering generic advice [53].

This mesosystemic disconnect channels help-seeking behaviours toward informal networks—primarily friends and family [54]. While these “strong ties” offer immediacy and familiarity, this reliance is more accurately an adaptation to a systemic void. Informal networks lack the professional capacity to address clinical distress. The core issue is a lack of functional integration: the formal system remains isolated, while informal systems are an inadequate substitute for professional care [55, 56], constituting a critical vulnerability in the student support ecology.

Why do students so strongly prefer informal peer support over formal mental health services? Our findings suggest three interlocking reasons. First, cultural factors play a dominant role. Mental health problems remain stigmatised in Chinese society, and seeking formal help may be perceived as “losing face”—a macrosystemic barrier that is discussed in detail in Sect.  5.4. Second, practical barriers within the university’s formal support system—lack of awareness, perceived ineffectiveness, and procedural hurdles—disconnect students from formal care, as detailed in our Results (Subtheme 2b). Third, peer support is perceived as more accessible, immediate, and non-judgmental, requiring no appointments or formal records. Cost was not a primary deterrent because the university’s counselling services were free. Thus, the preference for informal networks reflects a complex interplay of cultural stigma, structural disconnections, and the convenience of peer relationships.

### Exosystem: institutional logics and structural constraints

Our analysis reveals how rigid career pathways and paradoxical institutional policies collectively constrain students’ mental health. These external forces operate as pervasive background conditions, exacerbating pressures from more immediate ecological levels.

Participants reported that institutional systems designed for support often exhibited a structural paradox: their inherent design inadvertently created barriers. Outdated infrastructure hindered learning and posed safety risks. Administrative processes like chaotic course scheduling were perceived as counterproductive, adding burdens that consumed cognitive resources [57]. This misalignment underscores an exosystemic failure where institutional logic overrides student-centred well-being. Furthermore, competitive incentive structures were described as demotivating for students who perceived benchmarks as unattainably high, illustrating how mechanisms for promoting excellence can paradoxically foster disengagement [57].

One salient constraint was the perception of career development as a highly rigid path governed by external labour market demands. In participants’ accounts, an undergraduate degree was seen as insufficient, compelling students to pursue postgraduate degrees—“forced further study.” This creates a cycle of chronic stress, aligning with observations of “academic involution” [58]. Notably, this pressure extended beyond clinical medicine to other health professions, pointing to a potential misalignment between policy, training scales, and job market realities. The relentless push for credentials risks diverting focus from deep learning and authentic professional identity formation to narrow instrumentalism [59], subordinating individual aspirations to systemic demands. The RTME programme exemplifies an exosystemic force that directly configures students’ developmental trajectories. Based on the two RTME participants in this sample, it created a dynamic of “geographically constrained stability”—alleviating employment anxiety for one participant through guaranteed placement, while for the other it constituted a constraint that curtailed autonomy and created a central tension between security and aspiration. These findings are preliminary and require validation through larger, dedicated studies of RTME students.

### Macrosystem: the internalisation of macro-cultural values and behavioural guidance

At the macrosystem level, our analysis reveals how socio-cultural ideologies are internalised, shaping career motivations, mental health understanding, and help-seeking behaviours. These cultural scripts operate as pervasive forces that legitimise certain pathways while stigmatising others [60].

A key theme is the internalisation of a cultural script prioritising occupational stability and familial responsibility. Career choices in the health professions were heavily influenced by parental guidance and a societal preference for secure, state-sanctioned career paths, encapsulated in the “iron rice bowl” [61]. This script frames career choices as acts fulfilling familial expectations and ensuring long-term security.

Filial piety is a key mechanism through which family expectations influence career decisions. In the Chinese cultural context, students internalise parental aspirations as their own, as deviating from family wishes would be perceived as a failure of filial duty. This moral dimension adds psychological weight to career choices, making misaligned pathways particularly distressing [5]. This internalisation creates a complex psychological dynamic. While providing security and fulfilling filial duty, it can lead to significant internal conflict when personal interests diverge, potentially undermining intrinsic motivation and long-term professional engagement [24]. The macrosystemic value of “stability-first” thus functions as a dual-edged sword: it offers a socially validated path but may constrain individual autonomy and passion, crucial for professional identity formation and well-being [62, 63].

Students had absorbed macrosystemic values emphasising “toughness,” “self-reliance,” and “saving face” [64]. This process transforms external stigma into self-stigma, where psychological distress is interpreted as a personal failure [65].

In participants’ accounts, mental health was described as a matter of “personal digestion”, achieved through self-control rather than external assistance [66]. This internalised worldview creates a powerful, pre-emptive barrier to help-seeking. The fear of “losing face” is a potent deterrent [67, 68]. Consequently, students prioritised self-contained coping strategies, viewing formal help as a last resort. This pattern suggests that even with readily accessible services, this cultural script of self-sufficiency would likely suppress utilisation rates [69], highlighting a macrosystemic conflict where cultural values directly undermine preventive mental healthcare principles [70].

The concept of “face”, a culturally valued social image that individuals claim for themselves through interaction, adds another layer to understanding help-seeking reluctance [71]. In Chinese cultural contexts, seeking psychological help may be perceived as threatening one’s face because it publicly admits personal weakness or failure to self-manage emotional distress. This fear of “losing face” is compounded by the collective nature of face: an individual’s behaviour can bring shame not only to the person but also to their family (colloquially described as bringing shame to the clan). Consistent with this, research among Chinese medical students has shown that concerns about loss of face are significantly associated with more negative attitudes toward professional psychological help-seeking [72]. Within our participants’ accounts, the reluctance to seek formal help—even when distress was recognised—was implicitly shaped by these face-related concerns, reinforcing the macrosystemic barrier identified in our analysis.

### Chronosystem: chronological interactions and the reshaping of developmental trajectories

The chronosystem dimension emphasises the impact of temporal change, revealing that psychological distress is dynamically shaped by past experiences, academic transitions, and major socio-historical events, creating a life course of stress and resilience [73–75].

Participants’ well-being was influenced by a chronology of critical incidents interacting with academic phases. Discrete negative life events, such as exam failure or interpersonal conflict, had long-lasting effects, creating “psychological scars” that heightened vulnerability [76], aligning with life course theory [77].

Furthermore, participants’ accounts suggested an evolving pattern of stress across the academic journey. Early-stage students described challenges in adapting to autonomous learning, while senior students—particularly those in clinical internships—described intense pressure from balancing internship demands with postgraduate entrance examination preparation. However, given the small number of fifth-year clinical medicine students in our sample (*n* = 3), these observations regarding academic stage trajectories should be considered preliminary. This illustrates a chronosystemic pattern where stressors interact and intensify over time [78], suggesting interventions must be temporally sensitive.

The COVID-19 pandemic served as a powerful macrosystemic event that permeated all ecosystem levels, fundamentally altering the context of student development [79–81]. Academically, the shift to online learning decreased engagement, exacerbating isolation and anxiety [81].

More profoundly, the pandemic amplified pre-existing macrosystemic cultural values, particularly the prioritisation of career stability [23]. Familial pressure to choose “safe” professions intensified, indicating that major crises can accelerate the internalisation of cultural scripts. Additionally, the pandemic directly influenced career perceptions, with students in fields like preventive medicine perceiving a link between the pandemic’s emergence and future market saturation [82]. This shows how a chronosystemic event can influence the exosystemic job market dynamics, altering perceived trajectories [83]. The pandemic served as a catalyst for change, the full effects of which will continue to unfold over time [84].

### Limitations and future directions

This study provides a contextually rich, in-depth exploration of the ecological systems influencing the mental health of health professions students in China. While the qualitative methodology was essential for uncovering the complex, nuanced mechanisms across system levels, several aspects of the study’s design point to directions for future research.

First, the research was conducted at a single university in Jiangsu Province, a region with specific socioeconomic characteristics. This focused setting was crucial for achieving the analytical depth required for rigorous qualitative inquiry [85]. Although this may affect the direct transferability of findings to other contexts (e.g., vocational colleges or universities in less developed regions), the thick description provided allows readers to assess the potential relevance to their own settings [86]. Future research should test the ecological model proposed here through multi-institutional comparative studies across diverse geographic and socioeconomic contexts.

Second, the study’s reliance on participant self-report through interviews, while a strength for capturing lived experience, is inherently subjective. To mitigate potential biases (e.g., social desirability, recall bias) and enhance credibility, we employed rigorous methodological safeguards, including iterative questioning and a collaborative approach to coding and theme development to ensure interpretive validity [87]. Future studies could strengthen this line of inquiry through data triangulation, for instance, by incorporating direct observations of learning environments or analysing institutional policy documents to provide a more multi-faceted view of the ecological systems. In particular, our findings on faculty-student interactions are based on students’ self-reported perceptions, which may not fully capture the complexity of these communications or the faculty members’ intentions; classroom observations or interviews with faculty would provide a more complete picture.

Third, while our purposive sampling captured a diverse range of participants across year groups and programmes, the numbers of students in certain subgroups were small: fifth-year clinical medicine students (*n* = 3) and RTME programme students (*n* = 2). Consequently, our observations regarding academic stage trajectories and RTME experiences should be interpreted as preliminary and illustrative rather than as generalisable claims. Future research with larger, balanced samples across year groups and specific programme types is needed to further explore these patterns.

Fourth, we did not systematically collect data on participants’ socioeconomic backgrounds. While our sample included two RTME students (who are recruited from rural areas), we cannot comment on how family income or socioeconomic status may have shaped the experiences of the broader sample. Future research should examine whether students from lower socioeconomic status backgrounds experience different ecological pressures (e.g., financial stress, family dependence on future income) compared to their peers.

Finally, the qualitative design and sample size, determined by the principle of data saturation, were optimal for identifying the breadth and nature of influencing factors but not for measuring their prevalence or statistical associations. This prioritisation of explanatory depth over generalisable breadth is a recognised characteristic of qualitative research [27]. The themes generated, particularly those highlighting under-explored constructs such as the “structural paradox of institutional support” and “professional identity dissonance,” provide a robust foundational framework. A logical next step would be to develop and validate quantitative instruments based on these findings to empirically test the prevalence and interrelationships of these factors across larger, more representative populations.

## Conclusion

Guided by EST [26], this qualitative study elucidates the complex interplay of factors shaping the mental health of health professions students in China. Our analysis demonstrates that psychological distress arises from a dynamic transaction across the entire educational ecosystem. Immediate academic and interpersonal pressures (microsystem) are exacerbated by fragmented support networks (mesosystem), rigid institutional structures (exosystem), and the internalisation of cultural values that prioritise stability and stigmatise help-seeking (macrosystem), with these interactions evolving over time through critical events and transitions (chronosystem).

The primary contribution of this work is twofold. First, it provides crucial contextual depth to the global literature by detailing how universal ecological challenges manifest within China’s distinctive socio-cultural and institutional setting. Second, it offers a critical complementary perspective to dominant individual-level paradigms. By foregrounding the constitutive role of systemic forces, our findings argue for a necessary paradigm shift towards ecological explanations and interventions [19, 26].

Consequently, effectively supporting student well-being requires integrated, multi-level strategies. This entails: fostering supportive micro-environments through relational mentorship; bridging mesosystem gaps by embedding mental health support into academic life; reforming exosystem policies to create more flexible and humane developmental pathways; and initiating macrosystem dialogues to destigmatise psychological distress. Ultimately, cultivating a resilient future healthcare workforce demands a systemic response that aligns educational structures and cultural expectations with the fundamental goal of nurturing student mental health.

## Supplementary Information

Supplementary Material 1.

## Data Availability

The datasets used and/or analysed during the current study are available from the corresponding author on reasonable request. Any personally identifiable information within the dataset will not be disclosed.

## Abbreviations

CM: Clinical medicine
COREQ: Consolidated Criteria for Reporting Qualitative Research
COVID-19: Coronavirus Disease 2019
EST: Bronfenbrenner’s ecological systems theory
MLT: Medical laboratory technology
PM: Preventive Medicine
RTME: Rural-oriented Tuition-Waived Medical Education
