# Supplementary material for: Dual-edged swords: a qualitative study of protective and risk factors shaping mental health among Chinese health professions students
Source: BMC Med Educ. 2026 Aug 8;26:1789. doi: 10.1186/s12909-026-10111-2 (PMC13737903; doi:10.1186/s12909-026-10111-2)
Supplement: Supplementary file 1 [file 12909_2026_10111_MOESM1_ESM.docx]

**Interview Guide**

1. Learning Experience and Environmental Interaction

Opening Question: "Could you walk me through what a typical study week looks like for you here at the university?"

Follow-up (context & coping): "Can you tell me about a specific course, exam period, or time you found particularly demanding—what was that experience like for you, and how did you get through it?"

Follow-up (institutional impact): "Could you give some concrete examples of how a university policy, resource, or facility has affected your academic work or daily life—whether positively or negatively?"

2. Major Choice and Future Planning

Opening Question: "Would you feel comfortable sharing how you came to choose your current major?"

Follow-up (future plans): "What are your plans or aspirations for after graduation?"

Follow-up (reflection & connection): "Looking back now, how do you feel about your original major choice, and how does it connect to your current career plans?"

3. Coping Strategies and Support Networks

Opening Question: "When you face significant challenges in your academic or personal life, what do you find yourself usually doing in response?"

Follow-up (informal support): "Who among the people close to you—friends, roommates, or family—tends to be involved during difficult times? Can you recall a specific instance?"

Follow-up (formal support): "What is your awareness or impression of the formal support services offered by the university, such as psychological counselling or academic advising?"

4. Significant Experiences and Personal Development

Opening Question: "Looking back, even before university, what experiences—challenging or rewarding—do you feel have most shaped who you are today?"

Follow-up (university transitions): "How have the main pressures or challenges in your university life shifted from your first year until now?"

Follow-up (broader context): "In what ways, if at all, has any major external event—for example, the COVID-19 pandemic—influenced your approach to learning, your outlook on your career, or your personal goals?"
